# Capturing the clinical complexity in young people presenting to primary mental health services: a data-driven approach

**DOI:** 10.1017/S2045796024000386

**Published:** 2024-09-18

**Authors:** Caroline X. Gao, Nic Telford, Kate M. Filia, Jana M. Menssink, Sabina Albrecht, Patrick D. McGorry, Matthew Hamilton, Mengmeng Wang, Daniel Gan, Dominic Dwyer, Sophie Prober, Isabel Zbukvic, Myriam Ziou, Sue M. Cotton, Debra J. Rickwood

**Affiliations:** 1 Centre for Youth Mental Health, The University of Melbourne, Parkville, VIC, Australia; 2 Orygen, Parkville, VIC,Australia; 3 School of Public Health and Preventive Medicine, Monash University, Melbourne, VIC, Australia; 4 headspace, National Youth Mental Health Foundation, Melbourne, VIC, Australia; 5 Menzies Institute for Medical Research, University of Tasmania, Hobart, TAS, Australia; 6 Faculty of Health, University of Canberra, Canberra, ACT, Australia

**Keywords:** adolescent, complexity, episode of care, mental health, mental health services

## Abstract

**Aims:**

The specific and multifaceted service needs of young people have driven the development of youth-specific integrated primary mental healthcare models, such as the internationally pioneering *headspace* services in Australia. Although these services were designed for early intervention, they often need to cater for young people with severe conditions and complex needs, creating challenges in service planning and resource allocation. There is, however, a lack of understanding and consensus on the definition of complexity in such clinical settings.

**Methods:**

This retrospective study involved analysis of *headspace*’s clinical minimum data set from young people accessing services in Australia between 1 July 2018 and 30 June 2019. Based on consultations with experts, complexity factors were mapped from a range of demographic information, symptom severity, diagnoses, illness stage, primary presenting issues and service engagement patterns. Consensus clustering was used to identify complexity subgroups based on identified factors. Multinomial logistic regression was then used to evaluate whether these complexity subgroups were associated with other risk factors.

**Results:**

A total of 81,622 episodes of care from 76,021 young people across 113 services were analysed. Around 20% of young people clustered into a ‘high complexity’ group, presenting with a variety of complexity factors, including severe disorders, a trauma history and psychosocial impairments. Two moderate complexity groups were identified representing ‘distress complexity’ and ‘psychosocial complexity’ (about 20% each). Compared with the ‘distress complexity’ group, young people in the ‘psychosocial complexity’ group presented with a higher proportion of education, employment and housing issues in addition to psychological distress, and had lower levels of service engagement. The distribution of complexity profiles also varied across different *headspace* services.

**Conclusions:**

The proposed data-driven complexity model offers valuable insights for clinical planning and resource allocation. The identified groups highlight the importance of adopting a holistic and multidisciplinary approach to address the diverse factors contributing to clinical complexity. The large number of young people presenting with moderate-to-high complexity to *headspace* early intervention services emphasises the need for systemic change in youth mental healthcare to ensure the availability of appropriate and timely support for all young people.

## Introduction

Mental health disorders represent the predominant source of burden of disease among young people between the ages of 15 and 25 years (Gore *et al.*, 2011). Around 62.5% of mental disorders emerge by the age of 25 (Solmi *et al.*, 2022). Globally, 1 in 7 (14%) 10- to 19-year-olds have been estimated to experience mental ill-health (Global Burden of Disease; IHME, 2019). In Australia, young people aged 16–24 years exhibited the highest prevalence of mental health issues, with 45.5% of females and 32.4% of males having experienced a mental disorder in the past 12 months (ABS, 2023). Adolescence and early adulthood, marked by significant developmental changes, present diverse and complex mental health challenges influenced by family, environmental, health, social and economic factors (Patel *et al.*, 2007).

Despite the higher burden of mental ill-health and complex care needs, young people are less likely to seek help due to a range of factors, such as stigma and poor access to appropriate care (Rickwood *et al.*, 2007). To address these issues and minimise barriers to service access, an integrated youth-specific primary care model has been proposed (Hetrick *et al.*, 2017; McGorry *et al.*, 2019). The largest national implementation of this model, *headspace* services, was initiated in Australia in 2007. Presently, there are 154 *headspace* centres providing accessible, youth-friendly and holistic care for young people across all states and territories of Australia.

Although *headspace* was designed as a primary care service for mild-to-moderate mental health difficulties, it practices a ‘no wrong door’ policy (Rickwood *et al.*, 2019), ensuring young people are not turned away or left to navigate the mental health system alone. As a result, *headspace* has served as the primary point of care for young people with severe illnesses or complex needs, particularly those impacted by the ‘missing middle’ service gap who lack access to tertiary care (Menssink *et al.*, 2023). These individuals often present with severe symptoms and multiple challenges, including suicidality, frequent relapses, multiple diagnoses, cognitive issues, comorbidities, and social or vocational impairments (Orygen, 2021; Productivity Commission, 2020).

While primary care providers aim to assist young people with more complex needs by supporting transitions to tertiary or specialist care (Rickwood *et al.*, 2019), these efforts have been hindered by existing service gaps in the mental health system and workforce shortages. Young people with more complex factors presenting to primary services require specialised clinical expertise and more intensive and extended case management – including care coordination with external health and social care providers. This mismatch in services may result in heavier clinical workloads, increased service wait times and risk of under-treatment (headspace, 2019). Furthermore, providing services to more young people with greater complexity than the service is designed for also impacts service planning, resource needs and allocation, staffing needs and potentially treatment outcomes (Productivity Commission, 2020; State of Victoria, 2021).

Defining and measuring clinical complexity in youth primary care services remains challenging. The term ‘complexity’, although frequently used, lacks a clear and consistent definition in clinical settings (Hetrick *et al.*, 2011). In the adult mental health literature, complexity is historically viewed as the severity and persistence of mental illness (National Advisory Mental Health Council, 1993). Whiteford and colleagues, however, recognised this limitation and added a dimension from a needs perspective, to include ‘requiring multi-agency support to maximise their health, housing, social participation and personal functioning’ (Whiteford *et al.*, 2017). In the primary care context, complexity is often seen as a mix of health, psychosocial and environmental factors affecting care coordination (Manning and Gagnon, 2017; Safford *et al.*, 2007). However, these conceptualised definitions provide little guidance on how to operationalise measuring complexity in clinical settings to guide service planning and delivery. This is particularly concerning in youth mental health, where young people can present with a diverse range of factors associated with complexity (e.g., risks associated with illness, psychosocial stressors, lack of family support and interaction with the clinical workforce and service system) (Marama *et al.*, 2021) and the collective effects of these factors and how they interact with the service system and clinical workforce are largely unknown.

The comprehensive *headspace* minimum data set (MDS), collecting high-quality data on risk factors, outcomes and treatment engagement (Rickwood *et al.*, 2014), allows us to advance our understanding of client complexity which, in turn, can facilitate effective planning at both the service and individual treatment levels. In this study, we utilised modern ‘big data’ and machine learning approaches to develop a model-based definition of clinical complexity in youth-specific primary care settings. The aims of the study were to (i) explore complexity markers and their interrelationship in the routinely collected *headspace* MDS data and (ii) conduct exploratory clustering analysis to identify subgroups with different levels of complexity. Addressing these aims will allow us to better understand and articulate the nature of complexity of young people presenting to primary care youth mental health services in Australia. Findings will enable services to better plan and advocate for ways to accommodate the needs of all young people.

## Methods

### Study design and procedures

This is a retrospective study of a clinical treatment cohort of young people who started an episode of care (EOC) in *headspace* services between 1 July 2018 and 30 June 2019. The *headspace* is a youth-specific primary healthcare service in Australia for young people aged 12–25 years. It provides highly accessible, evidence-based, client-centred care to young people (McGorry *et al.*, 2019; Rickwood *et al.*, 2019). During the data collection period, 113 headspace centres were operating in Australia, delivering holistic care across the multiple domains of mental and physical health, sexual health, substance use and vocational engagement (Rickwood *et al.*, 2014). During the study, young people may have had multiple EOCs, which were analysed separately to account for potential changes in their circumstances and needs.

### Data collection and measures

The data used in the analyses were drawn from the *headspace* MDS collected from all young people and their service providers at initial assessment and service data from across their EOC (Rickwood *et al.*, 2014).

The MDS collects self-reported demographic information including age and gender identity, Aboriginal or Torres Strait Islander identity, sexuality, culturally and linguistically diverse (CALD) background, residential remoteness (using postcode information), and current education and employment engagement. As individual-level socioeconomic status was not collected as a part of MDS, clients’ residential postcodes were used as proxies for socio-economic status, applying the 2021 Index of Relative Socio-Economic Advantage and Disadvantage (IRSAD) ranking tertiles (ABS, 2021). IRSAD measures the socio-economic conditions of participant’s residential area, capturing both relative advantage (e.g., higher income, more skilled occupations) and disadvantage (e.g., lower income, higher unemployment).

Psychological distress was measured using the self-reported 10-item Kessler Psychological Distress Scale (K10; Kessler *et al.*, 2002). Total scores of K10 range from 10 to 50 with a score of 30 indicated very high psychological distress (Andrews and Slade, 2001). Overall quality of life was measured using the self-reported 5-item MyLifeTracker (MLT; Kwan *et al.*, 2018), which assessed young people’s satisfaction in the domains of general well-being, day-to-day activities, relationships with friends, relationships with family and general coping. MLT total scores range from 0 to 100 with higher scores indicating higher quality of life. Young people also self-reported the primary reason for contacting *headspace* and prior mental health service engagement, amidst other information pertinent to clinical care.

Primary and secondary diagnoses (including provisional) were established based on the Diagnostic and Statistical Manual of Mental Disorders (DSM-4; American Psychiatric Association, 1994) to align with the reporting requirements of the national Primary Mental Health Care Minimum Data Set (PMHC-MDS). Similarly, the stage of illness was evaluated by *headspace* clinicians to determine whether full- or sub-threshold criteria for a diagnosis was met. The staging model is comparable with the method proposed by McGorry *et al.* (2006), see Table S1 in Supplementary Material I.

At each visit, *headspace* clinicians reported on the young person’s primary presenting issues (e.g., current mental and physical health issues, situational issues, alcohol and other substance use issues, need for vocational assistance) and assess occupational and social functioning using the 100-point Social and Occupational Functioning Assessment Scale (SOFAS) (Goldman *et al.*, 1992). A SOFAS score of 60 or below indicates moderate-to-severe impairment in functioning. Finally, details regarding clinical services provided to young people, including service frequency, type and length were obtained from the headspace MDS.

### Mapping of complexity factors

Potential factors that may contribute to clinical complexity were identified and tested via four methods: firstly a narrative literature review which supported the development of a clinical practice point (Marama *et al.*, 2021), secondly identification by *headspace* clinical experts of complexity factors relevant to the *headspace* client group, thirdly having over 1200 *headspace* clinicians assess 11,120 young people against the identified complexity factors and fourthly interviewing 13 operational or clinical staff from across nine *headspace* centres to better understand the experience and impact of complexity at *headspace.* Through this comprehensive process, we identified a consistent range of factors including clinical stage of illness/severity, type of mental illness, illness history, level of distress, presence of comorbidities, suicidality, alcohol and drug use, impairment in functioning, developmental trauma/adverse childhood life experiences, developmental/cognitive difficulties, psychosocial stressors (e.g., homelessness, financial stressors, educational stressors, family issues), social disadvantage (e.g., educational, income, stigma, discrimination, isolation, poor mental health literacy) and physical disability/chronic health problems. These factors were mapped onto 13 clinical and non-clinical variables identified in the MDS (see definitions in Table S2). To maximise the amount of available information collected by the MDS over treatment visits, all 13 variables were extracted as binary risk factors and coded as ‘Yes’ when the risk factor was presented at assessments.

### Statistical analysis

Analyses were conducted using R version 4.2.2 (2022-10-31). Detailed procedures are provided in Supplementary Material II. In brief, we utilised simple descriptive statistics to examine the characteristics and complexity of a young cohort, including factors like age, gender and residence. Network analysis, specifically multidimensional scaling network plots, was used to visualise the interrelationships among complexity factors. To identify potential subgroups among all participants, we used clustering analysis. This unsupervised learning approach enables the identification of inherent patterns and subgroupings within the data, facilitating a deeper understanding of cohort heterogeneity (Gao *et al.*, 2023). We implemented K-means consensus clustering which integrates consensus clustering with multiple imputations, to overcome challenges related to missing data and stability issues of single-run clustering algorithms (Gao *et al.*, 2024; Wu *et al.*, 2015). The selection of the optimal number of clusters was based on clinical relevance, acknowledging limitations in model-driven methods for large samples. Multivariable multinomial logistic regression models were used to evaluate factors associated with complexity.

## Results

A total of 95,030 (EOCs from 88,004 young people were recorded between 1 July 2018 and 30 June 2019. Fourteen per cent (*n* = 13,408) of EOC were omitted from the study due to very high proportion of missing data (missing all complexity indicators). Comparisons of EOCs for excluded and included participants are provided in Table S3. The participant characteristics were broadly comparable except for the lower number of service uses among excluded EOCs. The analytic sample comprised a total of 81,622 EOC from 76,021 young people. The cohort covers diverse groups of young people who attended *headspace* services (see Table S4). Sixty per cent of young people identified as female and over half were aged under 18. Around 9% of participants reported being Aboriginal and/or Torres Strait Islander, and around 90% were from inner regional or major cities. On average, young people visited *headspace* four times across the year (Median 2, Q1–Q3: 1–5); these frequencies were distributed evenly across age groups. A slightly higher proportion of clients in younger age groups were from lower socio-economic status areas (38.6% of young people aged 12–14 were from the low IRSAD group compared with 27.7% among those aged 21–25, see Table S4).

### Complexity factors

**Figure 1.**
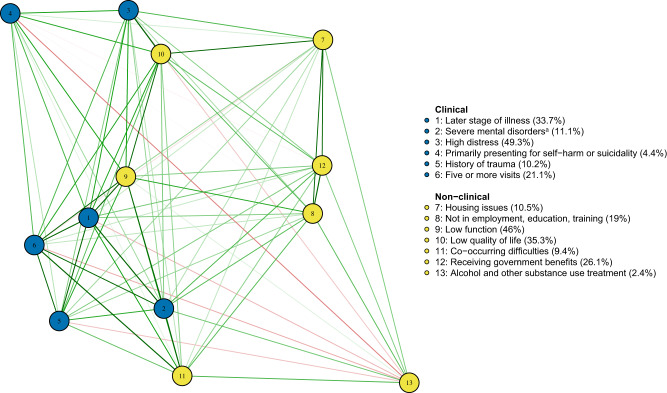
Network plot of complexity factors against treatment characteristics and other risk factors. Note: pairwise tetrachoric correlations (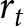) between complexity indicators were estimated from pooling 20 imputed datasets. ^a^Primary or secondary diagnosis of mental disorders with more complex needs (e.g., psychotic, bipolar, personality and neurodevelopmental disorders, see Table S2).

The prevalence of different complexity factors varied in the total population as well as in different age groups (see Table 1). Over 50% of clients reported experiencing very high psychological distress (K10 >30), and over 46% had low functioning (SOFAS score <61). Prevalence of later stage of illness and diagnoses of severe disorders (e.g., psychotic, bipolar and personality disorder) increased with age; however, clients aged 12–14 had the highest prevalence of self-harm or suicidality (6.2%) as the primary presenting issue. The proportion of clients with alcohol and other substance use as their primary issue for treatment was low. Mental health issues, particularly anxiety and depression, were the most common reasons for presentation. Network analysis suggested that most of the complexity indicators were positively associated (except for alcohol and other drug substance treatment, which had low prevalence), see Fig. 1 and S1. Strong associations were found between high distress and low quality of life, as well as between later stages of illness, severe disorders and low functioning. Housing issues also had a higher correlation with lack of engagement in employment, education or training, and with receiving government benefits.

**Table 1.** Profiles of individual complexity/risk factors by age group

	Age group					
	12−14 (*n* = 19,181)	15−17 (*n* = 24,406)	18−20 (*n* = 19,149)	21−25 (*n* = 18,719)	Total (*N* = 81622)	Missing
**Clinical factors**						
Later stage of illness	4951 (26.5%)	7723 (32.5%)	6891 (37.1%)	7281 (40.2%)	26,868 (33.9%)	2331
Severe mental disorders^a^	1486 (8.0%)	2310 (9.7%)	2404 (12.9%)	2663 (14.7%)	8872 (11.2%)	2338
High distress	6141 (38.3%)	10,501 (52.2%)	8786 (56.5%)	8194 (54.6%)	33,630 (50.4%)	14,891
Primary presenting for self-harm or suicidality	1162 (6.2%)	1110 (4.7%)	719 (3.9%)	549 (3.0%)	3540 (4.5%)	2434
History of trauma	1708 (9.1%)	2371 (10.0%)	1964 (10.6%)	2103 (11.6%)	8147 (10.3%)	2430
Five or more visits	4190 (21.8%)	5052 (20.7%)	4008 (20.9%)	3962 (21.2%)	17,224 (21.1%)	0
**Non-clinical risk factors**						
Housing issues	799 (5.0%)	1572 (7.9%)	2100 (13.5%)	1988 (13.3%)	6462 (9.7%)	15,068
Not in employment, education, training	1474 (9.2%)	2225 (11.1%)	4506 (28.9%)	4336 (28.8%)	12,547 (18.8%)	14,830
Low function	8660 (46.5%)	10,954 (46.3%)	8687 (46.9%)	8266 (45.8%)	36,602 (46.3%)	2631
Low quality of life	4363 (27.6%)	7277 (36.7%)	6240 (40.6%)	5896 (39.8%)	23,779 (36.1%)	15,747
Co-occurring difficulties	1469 (7.9%)	2164 (9.1%)	1967 (10.6%)	2012 (11.1%)	7615 (9.6%)	2335
Receiving government benefits	1350 (8.6%)	2691 (13.7%)	6140 (40.3%)	6440 (43.8%)	16,627 (25.5%)	16,402
Alcohol and other substance use treatment	79 (0.5%)	280 (1.5%)	340 (2.3%)	513 (3.6%)	1212 (1.9%)	17,658

a Primary or secondary diagnosis of mental disorders with more complex needs (e.g., psychotic, bipolar, personality and neurodevelopmental disorders, see Table S2).

### Clustering analysis

**Figure 2.**
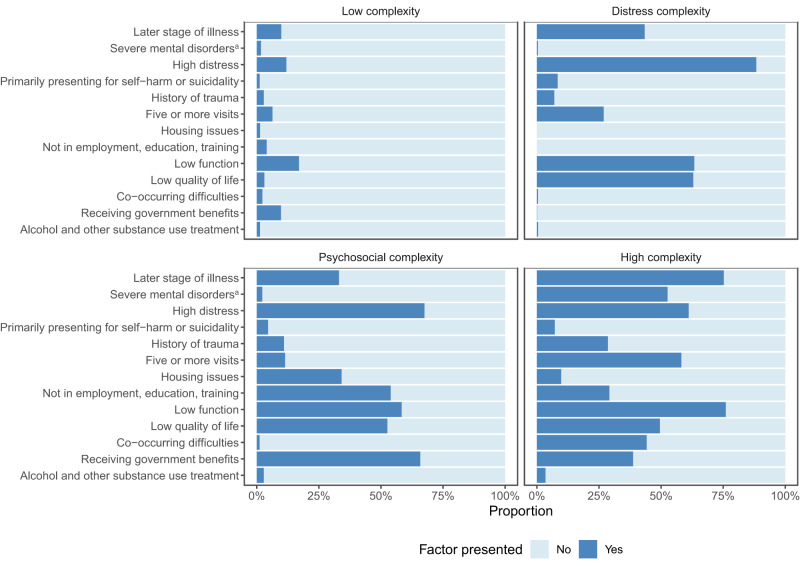
Results from 4-cluster solution: low complexity (*n* = 32,506, 39.8%); distress complexity (*n* = 16,251, 19.9%); psychosocial complexity (*n* = 17,781, 21.8%); high complexity (*n* = 15,084, 18.5%). Percentages of individual complexity factors in each subgroup are provided in Table S5. ^a^Primary or secondary diagnosis of mental disorders with more complex needs (e.g., psychotic, bipolar, personality and neurodevelopmental disorders, see Table S2).

The four-cluster solution, displayed in Fig. 2 and S2, provided the most informative segmentation of the client population (with clear variations in distributions of individual complexity factors, which can be interpreted clinically). Results from other solutions are provided in Fig. S3. Based on the number and types of complexity indicators presented in the four groups, around 40% of clients were classified as belonging to a group with a low level of complexity. The remaining three groups (two moderate – ‘distress complexity’ and ‘psychosocial complexity’ – and one high complexity, with ∼20% of clients per group) each showed distinctly different types and levels of complexity.

Clients in the ‘distress complexity’ cluster presented with higher levels of psychological distress, lower functioning and lower levels of quality of life than the ‘low complexity’ group. Clients in the ‘psychosocial complexity’ cluster, while similarly distressed, reported experiencing more psychosocial stressors including housing issues, not being in employment, issues with education or employment engagement, and reliance on government benefits. The ‘high complexity’ cluster represented the most severe cases with the highest proportion of clients presenting with a later stage of illness, serious disorders, history of trauma, low functioning and co-occurring difficulties (i.e., other issues such as physical, vocational, alcohol and substance use problems in combination with mental ill-health).

Client characteristics also differed substantially across clusters (see Table 2). Clients in the ‘low complexity’ group were slightly younger and a higher proportion identified as males. Clients in the ‘high complexity’ cluster had a higher proportion identifying as gender-diverse and a higher number of occasions of services (mean 7.1 visits per EOC). The ‘distress’ and ‘sychosocial complexity’ clusters also differed with more females in the ‘distress complexity’ and more males in the ‘psychosocial complexity’ cluster. Clients in the ‘psychosocial complexity’ cluster were older (mean age of 19), had a higher proportion of young people identified as Aboriginal and/or Torres Strait Islander and a higher proportion residing in inner regional areas. Furthermore, clients in the ‘psychosocial complexity’ cluster received a much lower level of treatment intensity (2.9 visits per EOC compared with 4.0 visits per EOC in the ‘distress complexity’ cluster).

**Table 2.** Characteristics of four cluster groups

	Low complexity *n* = 32,506 (39.8%)	Distress complexity *n* = 16,251 (19.9%)	Psychosocial complexity *n* = 17,781 (21.8%)	High complexity *n* = 15,084 18.5%)	*p*-value
**Gender**					<0.001
Female	15,989 (56.9%)	10,886 (71.0%)	9308 (59.2%)	8254 (57.5%)	
Male	11,855 (42.2%)	4217 (27.5%)	6134 (39.0%)	5642 (39.3%)	
Gender diverse	264 (0.9%)	240 (1.6%)	287 (1.8%)	448 (3.1%)	
**Age,** mean (SD)	16.8 (3.5)	16.6 (3.2)	19.0 (3.3)	18.1 (3.5)	<0.001
**Aboriginal and/or Torres Strait Islander**	2165 (7.9%)	1056 (6.9%)	1909 (12.4%)	1200 (8.4%)	<0.001
**Culturally and linguistically diverse^a^**	3062 (11.2%)	1676 (11.0%)	1619 (10.5%)	1454 (10.2%)	0.011
**Rurality**					<0.001
Major cities	19,670 (61.0%)	10,418 (64.5%)	10,733 (60.7%)	9215 (61.6%)	
Inner regional	8377 (26.0%)	4021 (24.9%)	5072 (28.7%)	4034 (27.0%)	
Outer regional	3479 (10.8%)	1564 (9.7%)	1660 (9.4%)	1443 (9.7%)	
Remote/very remote	699 (2.2%)	140 (0.9%)	188 (1.1%)	249 (1.7%)	
**Socio-economic status (IRSAD group)**					<0.001
Low	10147 (31.2%)	5395 (33.2%)	6597 (37.1%)	4871 (32.3%)	
Medium	10364 (31.9%)	5209 (32.1%)	5872 (33.0%)	5403 (35.8%)	
High	11973 (36.9%)	5645 (34.7%)	5304 (29.8%)	4800 (31.8%)	
**Total number of visits**					
Mean (SD)	2.3 (2.0)	4.0 (3.4)	2.9 (2.6)	7.1 (5.1)	<0.001
Median (Q1–Q3)	2 (1, 3)	3 (1, 6)	2.0 (1, 4)	6 (3, 9)	
**Number complexity factors presented^b^**					< 0.001
Mean (SD)	0.6 (0.7)	2.8 (1.1)	3.4 (1.7)	5.1 (1.9)	
Median (Q1–Q3)	1 (0, 1)	3 (2, 3)	3 (2, 4)	5 (4, 6)	

a Born in countries other than Australia and New Zealand or spoken language other than English at home.

b Number of complexity factors reported excluding missing data (missing data treated as not reporting the complexity factor).

Results from multivariable multinomial logistic regression are provided in Table 3. Young males presented with lower risk of being in the ‘distress’ and ‘psychosocial complexity’ cluster compared with young females, but comparable risk of being in the ‘high complexity’ cluster. Gender diverse identities remain the highest risk factor when comparing between groups. For example, the relative risk of being in the ‘high complexity’ group compared with the ‘low complexity’ group is 241% higher (relative risk ratio: 3.41, 95% confidence interval: 2.85–4.08) among gender diverse young people compared with young females. Young people identifying as Aboriginal and/or Torres Strait Islanders also had an 85% increase in relative risk of being in the ‘psychosocial complexity’ cluster, whereas being from another culturally or linguistically diverse background was a protective factor in the cohort. Different risk profiles were observed when the impact of socioeconomic status and remoteness were considered jointly. Living in higher socio-economic status areas is a protective factor for being in moderate-to-high complexity clusters. Nevertheless, when considering area-based socio-economic status and other risk factors, the likelihood of young people being in these higher complexity clusters decreases in more remote areas. This finding contrasts with the more evenly distributed crude data presented in Table 2.

**Table 3.** Multivariable multinomial logistic regression model results

	Distress vs. Low complexity		Psychosocial vs. Low complexity		High vs. Low complexity	
	RRR (95% CI)	*p*-value	RRR (95% CI)	*p*-value	RRR (95% CI)	*p*-value
**Gender**						
Female	Ref		Ref		Ref	
Male	0.54 (0.51–0.58)	<0.001	0.88 (0.84–0.92)	<0.001	0.93 (0.84–1.04)	0.204
Gender diverse	1.40 (1.16–1.69)	<0.001	1.90 (1.59–2.26)	<0.001	3.41 (2.85–4.08)	<0.001
**Age**	0.99 (0.98–0.99)	<0.001	1.22 (1.21–1.23)	<0.001	1.12 (1.12–1.13)	<0.001
**Aboriginal and/or Torres Strait Islander**						
No	Ref		Ref		Ref	
Yes	0.89 (0.72–1.09)	0.269	1.85 (1.44–2.37)	<0.001	1.16 (0.89–1.51)	0.282
**Culturally and linguistically diverse^a^**						
No	Ref		Ref		Ref	
Yes	0.60 (0.52–0.68)	<0.001	0.87 (0.78–0.98)	0.032	0.55 (0.52–0.58)	<0.001
**Rurality**						
Major cities	Ref		Ref		Ref	
Inner regional	0.80 (0.76–0.84)	<0.001	0.91 (0.87–0.96)	<0.001	0.88 (0.83–0.92)	<0.001
Outer regional	0.75 (0.70–0.80)	<0.001	0.72 (0.67–0.77)	<0.001	0.77 (0.72–0.83)	<0.001
Remote/very remote	0.35 (0.29–0.43)	<0.001	0.29 (0.24–0.35)	<0.001	0.55 (0.47–0.65)	<0.001
**Socio-economic status (IRSAD group)**						
Low	Ref		Ref		Ref	
Medium	0.94 (0.89–0.98)	0.007	0.81 (0.77–0.85)	<0.001	1.03 (0.98–1.09)	0.193
High	0.82 (0.78–0.87)	<0.001	0.52 (0.50–0.55)	<0.001	0.71 (0.67–0.75)	<0.001

*Note*: Missing data imputed using multiple imputation pooling estimates from 20 imputed datasets.

a Born in countries other than Australia and New Zealand or spoken language other than English at home.

CI = confidence interval; RR = relative risk ratio.

**Figure 3.**
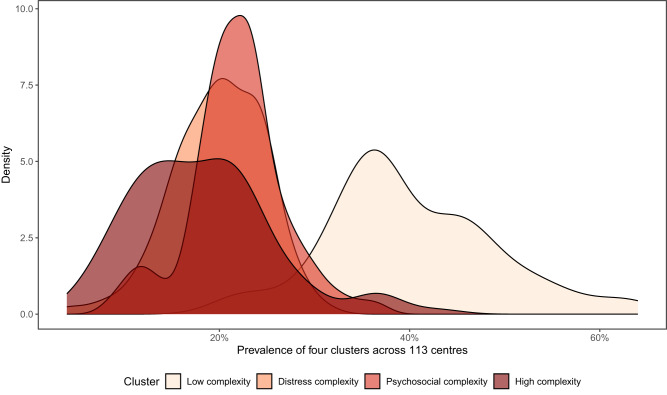
Smoothed distribution of young people’s complexity group prevalence across centres. The percentage of ‘Low Complexity’ group varied between 21% and 64% across centres, the ‘Distress Complexity’ group varied between 4% and 31%, the ‘Psychosocial Complexity’ group varied between 9% and 37% and the ‘High Complexity’ group varied between 4% and 43%.

The distributions of complexity clusters differ substantially across the 113 services providing care (Fig. 3). The proportions of clients in the high complexity clusters ranged from 4% to over 43% between centres. Similarly, the proportion of clients in the ‘low complexity’ cluster varied from 21% to 64%, indicating considerable local area diversity. Clients in moderate-to-high complexity groups tended to come from areas with lower socio-economic status.

## Discussion

While clinical complexity is crucial in care planning, there has been no clear consensus or model defining it. To the authors’ knowledge, this is the first paper to explore and attempt to conceptualise complexity using this approach in young people presenting for primary mental healthcare. We were able to differentiate clinical complexity profiles in young people by analysing 13 individual complexity factors using state-of-the-art consensus clustering methods. This data-driven approach suggested that clinical complexity can be constructed along two dimensions, comprising severity (low, moderate and high complexity) and type (distress versus psychosocial issues). The proposed complexity modelling approach provides much-needed direction to inform ways to manage clinical workload, undertake service and resource planning, bridge service gaps and improve clinical care for young people in integrated primary youth mental healthcare services.

Efforts to measure client complexity in youth mental healthcare remain scarce in the literature. Many existing complexity measures or tools were developed for adult services, such as community primary care (Pratt *et al.*, 2015; Shukor *et al.*, 2018), aged care (Boak *et al.*, 2024), internal medicine ward (de Jonge *et al.*, 2001) and community mental healthcare (Korasz *et al.*, 2018). Most of these tools rely on either self-report or clinical assessment, which adds additional strain to already limited clinical resources. Moreover, the domains mapped in these tools may not be relevant for youth mental health, such as maintaining lasting relationships and diagnosis uncertainty. Our study used data from a pre-established routine data collection process collected from a very diverse group of young people presenting to primary mental healthcare services across the country. Our results highlighted many domains relevant in delineating complexity subgroups, such as type of disorder, clinical stage, history of trauma, housing issues, co-occurring difficulties, financial security, as well as functioning. It is important to acknowledge that not all factors that potentially impact complexity are measured in this study (e.g., social isolation and lack of support). Our study presents foundational work supporting the development of a framework for measuring complexity in youth mental health settings.

The lack of consensus on clinical complexity has resulted in oversimplified tools for service planning and evaluation. For example, service needs are evaluated using only diagnosis and severity of illness in the Australian National Mental Health Service Planning Framework (AIHW, 2022). This approach cannot fully capture the nature of complexity for young people accessing primary, secondary and tertiary youth mental health services, nor does it support addressing the needs of young people in a holistic, biopsychosocial way.

In contrast, the complexity model we propose offers a multidimensional approach to understand the nature of complexity via the clustering of diverse but interconnected clinical and psychosocial factors. Our results highlight the need for a paradigm shift in how mental health complexity is understood and addressed for youth mental health services. This entails transitioning from a traditional clinical/diagnostic focus (e.g., diagnosis-related groups and case mix models) to a more holistic, multidimensional approach that considers the individual in their wider social and environmental context and facilitates cohesive care integration between primary and specialised services.

The issue of client complexity has emerged as an urgent challenge for youth mental health services like *headspace*, with centres maintaining that the complexity of young people accessing services is increasing and that they lack sufficient resources to attend to such needs (headspace, 2020). Our data confirm that approximately one in five young people presenting to *headspace* were clustered into the ‘high complexity’ subgroup. On average, these young people presented with five out of the 13 complexity factors evaluated, supporting the need for a holistic and multidisciplinary approach that integrates more specialised support with primary mental health services (Korasz *et al.*, 2018).

Note that our data were collected prior to the COVID-19 pandemic, which triggered significant and ongoing increases in mental ill-health among young people (ABS, 2023). The pandemic has also intensified the existing workforce shortages in mental health services, such as *headspace*, rendering the Medicare Benefits Schedule model unviable (KPMG, 2022) and impacting their ability to meet the demand (headspace, 2019). These challenges for services like *headspace* will continue to mount, and urgent reform in mental healthcare is needed (Armytage *et al.*, 2021; Productivity Commission, 2020).

Several risk factors, such as gender diversity, Aboriginal and/or Torres Strait Islander identity and socioeconomic status, were related to more complex clinical profiles, aligning with the social determinants of health lens in understanding complexity (Manning and Gagnon, 2017). This highlights the need for integrated services, with a ‘whole-person care’ approach, designed to adequately address other personal, social and environmental factors, in addition to clinical care needs (Bartholomeusz and Randell, 2022).

Importantly, there was substantial heterogeneity in the distribution of complexity subgroups across centres throughout the *headspace* network of services. Such variation is likely to be driven by many factors, including the specific needs in local areas, the availability of partnerships and the extent of integration with other services (e.g., different referral pathways and options) and capacity of the clinical workforce (e.g., some services have more experienced staff and can cater for the needs of more clinically complex clients). On other occasions, other stakeholders (e.g., service commissioners) may play a role in guiding client intake criteria. Our complexity model provides a much-needed way for centres to identify and evaluate the complexity of their clients, considering more than diagnosis and severity.

### Clinical implications

It is critical for clinical services to be able to be able to determine clinical complexity within their client group and subsequently prioritise and plan resourcing for care provision. At the clinician level, this is achieved via assessment and case formulation (Macneil *et al.*, 2012); however, views about client complexity within formulation vary among clinicians depending on their training, experience, clinical role and even their level of burnout (Manning and Gagnon, 2017). A data-driven framework, as we propose, can objectively quantify clinical resource needs. For instance, the capacity to consistently identify ‘high complexity’ young people presenting to care can assist service providers in tailoring interventions, prioritising resources to meet complex needs and liaising with enhanced care services and specialists. This data-driven model will be further developed to provide an easy-to-use checklist or automated algorithm that can be directly implemented into care.

Our findings show complexity is multifaceted. This highlights the need for a holistic care model supported by a multidisciplinary clinical team with considerable capacity (McGorry *et al.*, 2013). The distinction between the two moderate complexity groups (‘distress’ and ‘psychosocial complexity’) also reveals the essential need for broader psychosocial and community supports for those who require functional recovery, via programmes such as vocational interventions (e.g., Individual Placement and Support) (Joanna *et al.*, 2020; Simmons *et al.*, 2023), social prescribing (Drinkwater *et al.*, 2019) and housing and other supports (Ramsay *et al.*, 2011).

Variations in complexity profiles across different primary mental health services require further attention at the system financing level. The data-driven model we have developed can be used as the prototype to evaluate and understand different types of care models, as well as resourcing and staffing needs for an integrated national programme. For instance, a high prevalence of young people with ‘psychosocial complexity’ in specific services may indicate a greater demand for coordinating and supporting staff and educational and vocational support programmes. The findings also highlight that economic evaluations that do not consider complexity profiles (e.g., KPMG, 2022), and system financing models that do not account for how these profiles vary across centres, may not be sufficient or fit for purpose.

The identification of high proportions of young people with very complex profiles in primary mental health settings shows that further systemic change in youth mental healthcare is needed. The establishment of early psychosis services and, more recently, specialised youth-specific services provide directions for further expansion of early intervention models to other disorders (Patrick and Cristina, 2018), ideally via a transdiagnostic framework (McGorry *et al.*, 2022).

### Strengths, limitations and future directions

This study used a novel, integrated approach that combined clinical and data insights, and proposed a model that identifies and describes subgroups of young people with different levels of complexity. With rich clinical information collected from real-world nationwide clinical services, we were able to reduce the sampling and participation bias commonly observed in other study designs. We also used a state-of-the-art ensemble method to improve stability and big data scalability issues of clustering algorithms (Gao *et al.*, 2023).

The real-world data collected as a part of service operations may introduce bias due to missingness and accuracy in data collection. Ongoing improvement in data collection will assist further development of the concept and operation models of clinical complexity. Clustering models, as exploratory approaches, have limitations, including the possibility that results may change with different data sets as well as the choice of algorithm and parameters, leading to potential variability in subgroup identification. Temporal profiles in latent subgroups also require further evaluation. The current modelling framework involves characteristics associated with ongoing clinical care (e.g., diagnosis across multiple visits and number of visits).

Future research is needed to develop prediction models and clinical risk tools, using only baseline data, to support treatment and service planning when young people first present to care. It would also be of interest to determine whether complexity profiles affect young people’s experience of mental healthcare (Rickwood *et al.*, 2023) and the outcomes they achieve.

## Conclusion

We identified high proportions of young people with very complex profiles in primary mental health settings, which shows that further systemic change in youth mental healthcare is needed. The establishment of early psychosis services and, more recently, specialised youth-specific services provide directions for the expansion of early intervention models to other severe disorders (Patrick and Cristina, 2018), ideally via a transdiagnostic framework (McGorry *et al.*, 2022). It is our hope that this study leads to further discussion on the development of a unified approach for determining complexity in youth mental healthcare. This, in turn, will contribute to better planning and coordination, and ultimately to better care for young people.

## Supporting information

Gao et al. supplementary material Gao et al. supplementary material

## Data Availability

The data used in this study are not available publicly as they are derived from a routinely collected minimum data set that contains confidential and sensitive information and has strict privacy and consent restrictions. Analysis codes of this study are openly available online (https://osf.io/39gva/).

## Supplementary material

The supplementary material for this article can be found at https://doi.org/10.1017/S2045796024000386.
