# Supplementary material for: Capturing the clinical complexity in young people presenting to primary mental health services: a data-driven approach
Source: Epidemiol Psychiatr Sci. 2024 Sep 18;33:e39. doi: 10.1017/S2045796024000386 (PMC11450420; doi:10.1017/S2045796024000386)
Supplement: Gao et al. supplementary material [file S2045796024000386sup001.docx]

# Supplementary Material I: Supplementary tables and figures

Table S1. Clinical staging model summary

| **Clinical stage** | **Definition** |
| --- | --- |
| **Stage 0** | No symptoms of mental health problems or disorder. |
| **Stage 1a** | Mild to moderate general symptoms of mental health problems and/or high-risk psycho-social stressors (i.e., bullying, relationship problems). |
| **Stage 1b** | Symptoms that may indicate a diagnosable DSM mental disorder, but do not meet full threshold for diagnosis. |
| **Stage 2** | Full threshold DSM diagnosis. |
| **Stage 3** | Period of remission from full threshold diagnosis. |
| **Stage 4** | Ongoing severe symptoms of diagnosed disorder with no asymptomatic periods. |
| **Not Applicable** | If the young person is accessing headspace solely for a **non-mental health related issue** (i.e., physical health issue, vocational support, with no indication of mental health problems) or you are completely unable to adequately assess their stage of illness. |

Table S2. Identified clinical and other non-clinical risk factors associated with complexity

| **Label** | **Source** |
| --- | --- |
| **Clinical factors** | |
| The later stage of illness | Clinical staging 2+ |
| Severe mental disorders | Primary or secondary diagnosis of more severe mental health disorders including psychotic, bipolar, personality, obsessive, dissociative, substance use, paraphilic, neurocognitive, neurodevelopmental disorders, sexual disfunctions and other disorders related to adverse effects of medication use. General anxiety and depression were excluded. |
| High distress | K10>30 |
| Primary presenting issue of self-harm or suicidality | Clinician reported a primary presenting issue of deliberate self-harm, suicidal thoughts, or behaviour |
| History of trauma | Clinician reported a primary presenting issue of trauma or diagnosed with (primary or second) PTSD |
| Five or more visits | Over 5 treatment sessions for the current EOC |
| **Non-clinical factors** | |
| Housing issue | Self-reported homelessness or unstable living situation |
| NEET | Not in employment, education or training |
| Low function | SOFAS<61 |
| Low quality of life | MLT<37.5 |
| Co-occurring difficulties | Reported primary issues of being mental health or situational in combination of at least one additional other domain (physical health, sexual health, vocational issues, alcohol and substance use) across multiple occasion of services. |
| Receiving government benefits | Self-reported currently receiving government benefits |
| Alcohol and other substance use treatment | Clinician reported primary issue of alcohol and other substance or treated for substance abuse |

Table S3. Characteristics of young people across EOCs included and excluded in the analysis

|  | **Excluded (n=13408)** | **Included (n=81622)** | **p value** |
| --- | --- | --- | --- |
| **Age at the start of EOC** | |  | < 0.001 |
| Mean (SD) | 17.8 (3.5) | 17.5 (3.5) |  |
| Median (Q1, Q3) | 18.0 (15.0, 20.0) | 17.0 (15.0, 20.0) |  |
| Missing | 214 | 167 |  |
| **Gender** | |  | < 0.001 |
| Female | 3659 (56.2%) | 44437 (60.4%) |  |
| Male | 2747 (42.2%) | 27848 (37.9%) |  |
| Gender diverse | 108 (1.7%) | 1239 (1.7%) |  |
| Missing | 6894 | 8098 |  |
| **Aboriginal and/or Torres Strait Islander** | |  | < 0.001 |
| No | 4808 (81.6%) | 66029 (91.3%) |  |
| Yes | 1084 (18.4%) | 6330 (8.7%) |  |
| Missing | 7516 | 9263 |  |
| **Culturally and linguistically diverse^*^** | |  | < 0.001 |
| No | 4952 (85.5%) | 64504 (89.2%) |  |
| Yes | 841 (14.5%) | 7811 (10.8%) |  |
| Missing | 7615 | 9307 |  |
| **Rurality** | |  | < 0.001 |
| Major Cities of Australia | 7323 (55.4%) | 50036 (61.8%) |  |
| Inner Regional Australia | 4036 (30.5%) | 21504 (26.6%) |  |
| Outer Regional Australia | 1609 (12.2%) | 8146 (10.1%) |  |
| Remote/very remote Australia | 248 (1.9%) | 1276 (1.6%) |  |
| Missing | 192 | 660 |  |
| **Total number of visits** | |  | < 0.001 |
| Mean (SD) | 1.3 (1.4) | 3.7 (3.6) |  |
| Median (Q1, Q3) | 1.0 (1.0, 1.0) | 2.0 (1.0, 5.0) |  |
| Missing | 0 | 0 |  |
| **Socio-economic status (IRSAD group)** | |  | < 0.001 |
| Low | 4650 (34.7%) | 27010 (33.1%) |  |
| Medium | 4199 (31.3%) | 26848 (32.9%) |  |
| High | 4554 (34.0%) | 27722 (34.0%) |  |
| Missing | 5 | 42 |  |

^*^ Born in countries other than Australia and New Zealand or spoken language other than English at home.

Table S4. Characteristics of young people across all EOCs

|  | Age group | | | | Total  (N= 81622) |
| --- | --- | --- | --- | --- | --- |
|  | **12-14 (N=19181)** | **15-17 (N=24406)** | **18-20(N=19149)** | **21-25 (N=18719)** |  |
| **Gender identity** |  |  |  |  |  |
| Female | 10376 (59.9%) | 13736 (62.5%) | 10543 (60.9%) | 9761 (57.9%) | 44437 (60.4%) |
| Male | 6708 (38.7%) | 7875 (35.8%) | 6463 (37.3%) | 6784 (40.2%) | 27848 (37.9%) |
| None | 235 (1.4%) | 368 (1.7%) | 315 (1.8%) | 321 (1.9%) | 1239 (1.7%) |
| **Aboriginal and/or Torres Strait Islander** | 1959 (11.5%) | 2047 (9.5%) | 1235 (7.2%) | 1085 (6.5%) | 6330 (8.7%) |
| **Culturally and linguistically diverse^*^** | 1658 (9.8%) | 2228 (10.3%) | 1873 (11.0%) | 2046 (12.3%) | 7811 (10.8%) |
| **Rurality of young people’s residential address** | | |  |  |  |
| Major Cities | 11063 (58.2%) | 14593 (60.2%) | 12109 (63.7%) | 12121 (65.2%) | 50036 (61.8%) |
| Inner Regional | 5365 (28.2%) | 6647 (27.4%) | 4949 (26.0%) | 4531 (24.4%) | 21504 (26.5%) |
| Outer Regional | 2345 (12.3%) | 2619 (10.8%) | 1644 (8.6%) | 1536 (8.3%) | 8146 (10.1%) |
| Remote/very Remote | 227 (1.2%) | 342 (1.4%) | 308 (1.6%) | 396 (2.1%) | 1276 (1.6%) |
| **Socio-economic status (IRSAD group)** |  |  |  |  |  |
| Low | 7397 (38.6%) | 8671 (35.5%) | 5746 (30.1%) | 5180 (27.7%) | 27010 (33.1%) |
| Medium | 6545 (34.1%) | 7984 (32.7%) | 6322 (33.1%) | 5963 (31.9%) | 26848 (32.9%) |
| High | 5239 (27.3%) | 7749 (31.8%) | 7049 (36.9%) | 7568 (40.4%) | 27722 (34.0%) |
| **Total number of visits** |  |  |  |  |  |
| Mean (SD | 3.7 (3.5) | 3.6 (3.6) | 3.6 (3.8) | 3.7 (3.7) | 3.7 (3.6) |
| Median (Q1-Q3) | 2 (1, 5) | 2 (1, 5) | 2 (1, 5) | 2 (1, 5) | 2 (1, 5) |

Note: The dataset contains 167 missing values for age, 8098 for gender identity, 9263 for Aboriginal and/or Torres Strait Islander identity, 9307 for CALD, 660 for rurality and 42 for IRSAD group; ^*^Born in countries other than Australia and New Zealand or spoken language other than English at home.

Table S5: Complexity subgroups in the 4 cluster solutions

|  | **Low  complexity**  **n=32506 (39.8%)** | **Distress  complexity**  **n=16251 (19.9%)** | **Psychosocial  complexity n=17781 (21.8%)** | **High  complexity n=15084 (18.5%)** |
| --- | --- | --- | --- | --- |
| **Clinical factors** |  |  |  |  |
| **Later stage of illness** | 3107 (9.9%) | 6797 (43.4%) | 5657 (33.1%) | 11307 (75.2%) |
| **Severe mental disorders^*^** | 531 (1.7%) | 60 (0.4%) | 376 (2.2%) | 7905 (52.6%) |
| **High distress** | 2919 (11.9%) | 12930 (88.3%) | 9540 (67.5%) | 8241 (61.1%) |
| Primary presenting for self-harm or suicidality | 368 (1.2%) | 1310 (8.4%) | 781 (4.6%) | 1081 (7.2%) |
| **History of trauma** | 883 (2.8%) | 1098 (7.0%) | 1874 (11.0%) | 4292 (28.6%) |
| **Five or more visits** | 2061 (6.3%) | 4369 (26.9%) | 2024 (11.4%) | 8770 (58.1%) |
| **Non-clinical risk factors** |  |  |  |  |
| **Housing issues** | 324 (1.3%) | 0 (0.0%) | 4832 (34.2%) | 1306 (9.8%) |
| **Not in employment, education, training** | 974 (4.0%) | 0 (0.0%) | 7637 (53.9%) | 3936 (29.2%) |
| **Low function** | 5315 (17.0%) | 9920 (63.4%) | 9938 (58.3%) | 11429 (76.0%) |
| **Low quality of life** | 740 (3.1%) | 9121 (63.0%) | 7312 (52.6%) | 6606 (49.5%) |
| **Co-occurring difficulties** | 707 (2.2%) | 67 (0.4%) | 196 (1.1%) | 6645 (44.2%) |
| **Receiving government benefits** | 2313 (9.8%) | 8 (0.1%) | 9106 (65.8%) | 5200 (38.7%) |
| Alcohol and other substance use treatment | 306 (1.3%) | 67 (0.5%) | 379 (2.8%) | 460 (3.5%) |

^*^Primary or secondary diagnosis of mental disorders with more complex needs (e.g., psychotic, bipolar, personality and neurodevelopmental disorders, see Table S2)

Fig. S1. Pairwise tetrachoric correlations ($r_{t}$) between complexity indicators. Note: Pairwise tetrachoric correlations ($r_{t}$) between complexity indicators were estimated from pooling 20 imputed datasets. The dendrogram indicates clusters of variables estimated using hierarchical clustering with complete linkage. ^*^Primary or secondary diagnosis of mental disorders with more complex needs (e.g., psychotic, bipolar, personality and neurodevelopmental disorders, see Table S2)


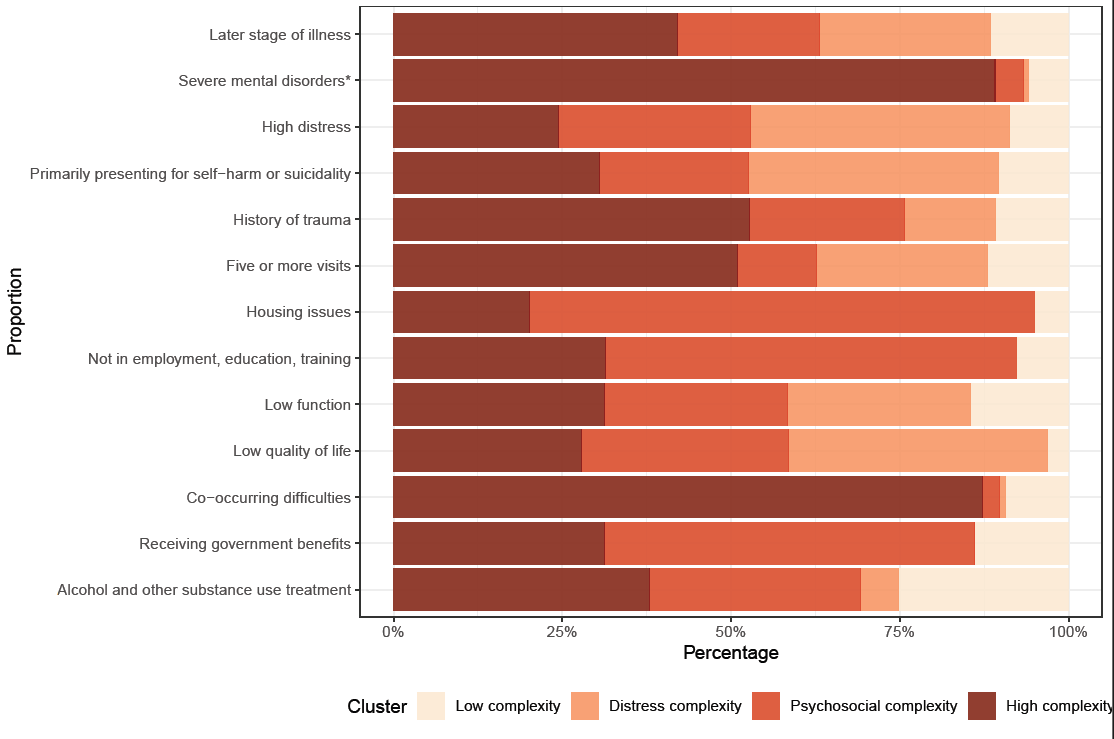

Fig. S2. Distribution of individual complexity factor by cluster in the 4 cluster solutions: low complexity (n=32506, 39.8%); distress complexity (n=16251, 19.9%); psychosocial complexity (n=17781, 21.8%); high complexity (n=15084, 18.5%). ^*^Primary or secondary diagnosis of mental disorders with more complex needs (e.g., psychotic, bipolar, personality and neurodevelopmental disorders, see Table S2)


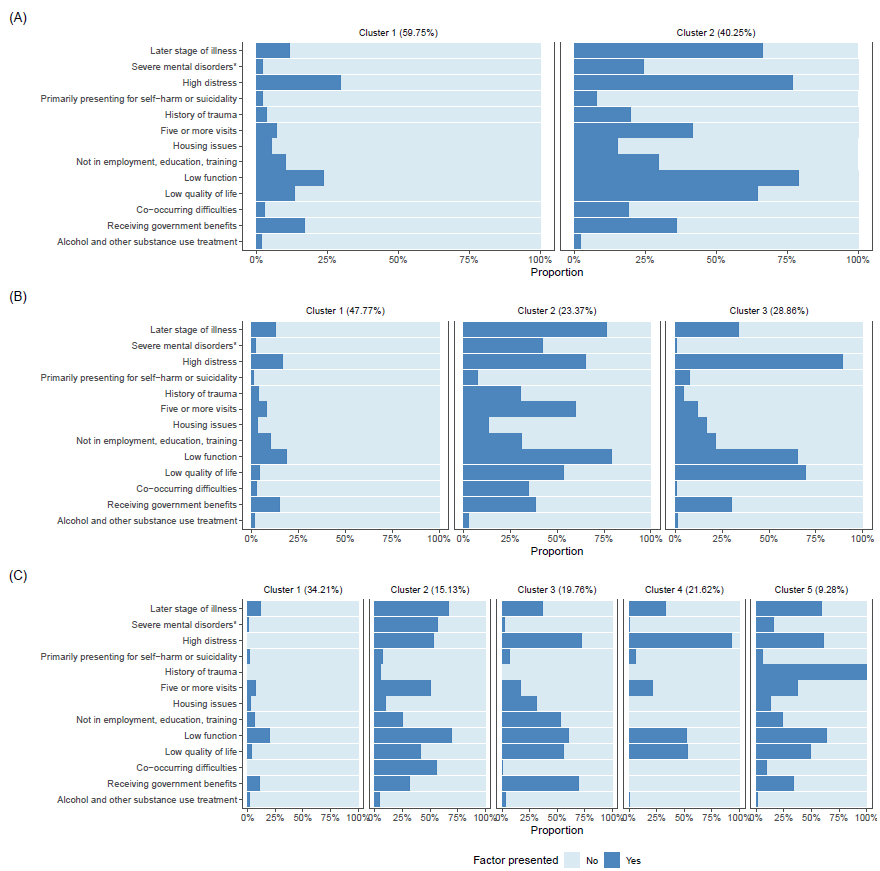


Fig. S3. Results from (A) 2-cluster, (B) 3-cluster, and (C) 5-cluster solutions.

# Supplementary Material II: Detailed statistical analysis methods

### Basic descriptive statistics

Simple descriptive statistics were used to explore the characteristics of the cohort and determine the respective proportions of young people presenting with different types of complexity indicators as well as associated risk factors (e.g., age, gender, area of residence).

### Network analysis

A multidimensional scaling network plot was employed to illustrate the interrelations among complexity factors, visualising the pairwise tetrachoric correlations ($r_{t}$) between them. Network plots summarise the multidimensional pairwise associations in a two-dimensional space, with nodes representing variables, and links between nodes representing their associations. It has a direct graphical interpretation, with shorter distances representing stronger associations, and thus providing a tangible picture of the overall connectivity between variables (Jones et al. 2018). Due to the high proportion of missing data in a few indicators, $r_{t}$ was estimated as an aggregated value from 20 imputed datasets, using multiple imputation by chained random forest (using R function missRanger) (Mayer 2021).

### Clustering

Consensus clustering was used to improve clustering model stability and performance (Fred and Jain 2002; Fred and Jain 2005; Monti et al. 2003; Strehl and Ghosh 2002; Topchy et al. 2004). We chose to use k-means clustering due to its high scalability to large datasets. However, results for k-means clustering can be impacted by the random starting point, and the k-means clustering algorithm cannot account for missing data (Gao et al. 2023). Therefore, we implemented k-means based consensus clustering on imputed datasets, as proposed by Wu and colleagues (Wu et al. 2015). To increase the consensus clustering performance, we further introduced the diversity of base clusters by randomly selecting 8 out of 13 clustering variables 10 times from each of the 20 imputed datasets, forming 200 base cluster datasets. We ran multiple correspondence analysis (MCA) for each base cluster dataset, extracting the first five dimensions. K-means clustering was then carried out on the Euclidean distance calculated from the MCA dimensions to generate 200 base clusters. The final clustering result was obtained by estimating a consensus using k-means clustering on the binary cluster association matrix (each cluster label converged to multiple binary variables representing individual partitions) generated from the 200 base cluster labels (Wu et al. 2015). Selecting the optimal number of clusters for consensus clustering remains challenging with model-driven methods. Evidence indicates that widely-used stability-based measures (e.g., (Monti et al. 2003; Șenbabaoğlu et al. 2014)) tend to underperform in some scenarios involving well-separated clusters (He and Yu 2019). Moreover, the computational cost required to calculate the consensus matrix and stability indices escalates exponentially as the sample size grows. Due to the explorative nature of the study and the large sample size, the best number of clusters was determined based on their clinical relevance.

Differences in the characteristics of young people among identified clusters were then compared using chi-squared (χ^2^) tests for categorical variables and analyses of variance (ANOVA) for continuous variables. Multivariable multinomial logistic regression models were used to evaluate whether these factors independently contributed to variations across different clusters. Missing data were addressed using the multiple imputed data with Relative Risk Ratio (RRR) pooled using Rubin’s rules (Rubin 2004).

### Reference

**Fred ALN and Jain AK** (2002) Data clustering using evidence accumulation. In: *2002 International Conference on Pattern Recognition.*

**Fred ALN and Jain AK** (2005) Combining multiple clusterings using evidence accumulation. *IEEE Transactions on Pattern Analysis and Machine Intelligence* **27**(6)**,** 835-850. <https://doi.org/https://doi.org/10.1109/TPAMI.2005.113>.

**Gao CX, Dwyer D, Zhu Y, Smith CL, Du L, Filia KM, Bayer J, Menssink JM, Wang T, Bergmeir C, Wood S and Cotton SM** (2023) An overview of clustering methods with guidelines for application in mental health research. *Psychiatry Research* **327,** 115265. <https://doi.org/10.1016/j.psychres.2023.115265>.

**He Z and Yu C** (2019) Clustering stability-based Evolutionary K-Means. *Soft Computing* **23**(1)**,** 305-321. <https://doi.org/10.1007/s00500-018-3280-0>.

**Jones PJ, Mair P and McNally RJ** (2018) Visualizing Psychological Networks: A Tutorial in R. *Frontiers in Psychology* **9**(1742). <https://doi.org/10.3389/fpsyg.2018.01742>.

**Mayer M** (2021) missRanger: Fast imputation of missing values. Available at <https://cran.r-project.org/web/packages/missRanger/missRanger.pdf> (accessed.

**Monti S, Tamayo P, Mesirov J and Golub T** (2003) Consensus Clustering: A Resampling-Based Method for Class Discovery and Visualization of Gene Expression Microarray Data. *Machine Learning* **52**(1)**,** 91-118. <https://doi.org/https://doi.org/10.1023/A:1023949509487>.

**Rubin DB** (2004) *Multiple imputation for nonresponse in surveys*. John Wiley & Sons.

**Șenbabaoğlu Y, Michailidis G and Li JZ** (2014) Critical limitations of consensus clustering in class discovery. *Scientific Reports* **4**(1)**,** 6207. <https://doi.org/https://doi.org/10.1038/srep06207>.

**Strehl A and Ghosh J** (2002) Cluster ensembles - a knowledge reuse framework for combining multiple partitions. *Journal of machine learning research* **3**(Dec)**,** 583-617. <https://doi.org/https://doi.org/10.1162/153244303321897735>.

**Topchy A, Jain AK and Punch W** (2004) A mixture model for clustering ensembles. In: *Proceedings of the 2004 SIAM international conference on data mining.* SIAM.

**Wu J, Liu H, Xiong H, Cao J and Chen J** (2015) K-Means-Based Consensus Clustering: A Unified View. *IEEE Transactions on Knowledge and Data Engineering* **27**(1)**,** 155-169. <https://doi.org/https://doi.org/10.1109/TKDE.2014.2316512>.
